# Pronounced peptide selectivity for melanoma through tryptophan end-tagging

**DOI:** 10.1038/srep24952

**Published:** 2016-04-27

**Authors:** Dinh Thuy Duong, Shalini Singh, Mojtaba Bagheri, Navin Kumar Verma, Artur Schmidtchen, Martin Malmsten

**Affiliations:** 1 Lee Kong Chian School of Medicine, Nanyang Technological University, 11 Mandalay Road, Singapore 308232; 2 Department of Pharmacy, Uppsala University, SE-75123, Uppsala, Sweden; 3 Division of Dermatology and Venereology, Department of Clinical Sciences, Lund University, SE-221 84 Lund, Sweden

## Abstract

Effects of oligotryptophan end-tagging on the uptake of arginine-rich peptides into melanoma cells was investigated under various conditions and compared to that into non-malignant keratinocytes, fibroblasts, and erythrocytes, also monitoring resulting cell toxicity. In parallel, biophysical studies on peptide binding to, and destabilization of, model lipid membranes provided mechanistic insight into the origin of the selectivity between melanoma and non-malignant cells. Collectively, the results demonstrate that W-tagging represents a powerful way to increase selective peptide internalization in melanoma cells, resulting in toxicity against these, but not against the non-malignant cells. These effects were shown to be due to increased peptide adsorption to the outer membrane in melanoma cells, caused by the presence of anionic lipids such as phosphatidylserine and ganglioside GM1, and to peptide effects on mitochondria membranes and resulting apoptosis. In addition, the possibility of using W-tagged peptides for targeted uptake of nanoparticles/drug carriers in melanoma was demonstrated, as was the possibility to open up the outer membrane of melanoma cells in order to facilitate uptake of low Mw anticancer drugs, here demonstrated for doxorubicin.

Cationic amphiphilic peptides have attracted considerable recent attention in both academic research and industrial drug development. Much of the increased interest in such peptides has been generated by antimicrobial peptides (AMPs), which play a key role in host defense. Through direct membrane lysis, these peptides provide fast and broad-spectrum antimicrobial effects, in some cases also for pathogens displaying resistance to conventional antibiotics^1^,^2^. It is also becoming increasingly recognized that some AMPs display additional host defense properties, including anti-inflammatory and immune modulating effects, of interest, e.g., for preventing sepsis development^3^,^4^. Some AMPs have furthermore been found to display anticancer effects, which are thought to originate from membrane destabilization of either cancer cells or cell compartments^5^,^6^,^7^,^8^. A key issue for the use of cationic amphiphilic peptides in this context is therefore that of selectivity, so that membranes of cancer cells are destabilized, but not those of healthy cells.

The specificity of cationic amphiphilic peptides to cancer cells seems to originate from differences in membrane composition between cancer cells and non-malignant ones. Notably, a wide range of cancer cells display higher content of anionic membrane components, notably phosphatidylserine (PS), sialic acid (linked to, e.g., glycoproteins and glycolipids), and heparan sulfate, while non-cancer cells exhibit an overall neutral charge due to zwitterionic phosphatidylcholine and sphingomyelin^5^,^6^,^7^,^8^. In fact, the occurrence of outer leaflet PS in a wide range of cancer types is so general that it has been suggested as a cancer marker in diagnostics. Similarly, sialic acid is broadly up-regulated in cancer cells. Indeed, the degree of sialyation has been found to correlate to the metastatic potential of such cells^9^,^10^,^11^. Amplifying such compositional differences, cancer cells have been found to form an increased number of microvilli, in turn resulting in a larger cell surface area, making them more susceptible to cationic amphiphilic peptides^7^,^12^.

In our previous work, we identified W- and F-tagging of AMPs as a means to provide potent broad-spectrum antimicrobial activities, also against various clinical isolates and multi-resistant strains, including vancomycin-resistant enterococci, multi-drug resistant *P. aeruginosa*, and methicillin-resistant *S. aureus*^13^,^14^,^15^. Importantly, such peptides display simultaneously low toxicity against eukaryotic cells, e.g., allowing both Gram-positive and Gram-negative bacteria to be effectively killed in blood at essentially non-existent hemolysis. Given the importance of electrostatic and lateral elastic interactions for this selectivity, discussed previously^16^, as well as the up-regulation of anionic lipids such as phosphatidylserine and gangliosides in a wide range of tumor cells, we hypothesized that the extreme selectivity displayed by some W-tagged peptides may translate also to selectivity between cancer cells and non-malignant ones.

For this purpose, we identified melanoma as a cancer type of particular interest. Thus, melanoma is a skin cancer that spreads earlier and more aggressively than other skin cancers. Together with limitations with current chemotherapies related to side-effects and chemoresistance development, this makes metastatic melanoma the most malignant skin cancer, accounting for >65% of all skin cancer-related deaths^17^. In quantitative terms, the American Cancer Society recently projected about 73,870 new melanomas to be diagnosed during 2015 in the US alone, resulting in some 9,940 deaths^18^. Due to this, there is an urgent need to identify novel therapeutic drug candidates.

Consequently, effects of W-tagging on peptide uptake into different melanoma cells was investigated under various conditions with confocal microscopy and flow cytometry, and compared to that into non-malignant fibroblasts and keratinocytes, also monitoring resulting toxicity by several different assays. In parallel, biophysical studies on peptide binding to, and destabilization of, model lipid membranes provided mechanistic insight into the origin of the selectivity between melanoma and non-malignant cells. The latter included also studies of mitochondria-mimicking model membranes, thereby providing information of the relative importance of outer membrane destabilization and facilitated apoptosis through mitochondria membrane destabilization. In addition, the possibility of using adsorbed W-tagged peptides for targeting of nanoparticles/drug carriers to melanoma cells was demonstrated, as was the possibility to open up the outer membrane of melanoma cells in order to facilitate uptake of low Mw anticancer drugs and improved anticancer effects, here demonstrated for doxorubicin.

## Experimental

### Chemicals

GRR10 (GRRPRPRPRP), GRR10-Alexa488 (GRRPRPRPRP-Alexa488), GRR10W4 (GRRPRPRPRPWWWW), and GRR10W4-Alexa488 (GRRPRPRPRPWWWW-Alexa488) were all synthesized by Biopeptide Co. (San Diego, USA), and were of >95% purity, as evidenced by mass spectral analysis (MALDI-TOF Voyager). Doxorubicin hydrochloride (>98%) was from Sigma (St. Louis, USA), as was carboxylated polyacrylonitrile-based nanoparticles (Chromeon 470-marked). All other chemicals used were of analytical quality.

### Cell culture

RPMI 7951 and MeWo human melanoma cell lines were obtained from ATCC, while HaCat cells and fibroblasts were obtained from AddexBio and ATCC, respectively. Cells were cultured in DMEM medium (Sigma-Aldrich) with 10% fetal bovine serum and grown at 37 °C in a humidified incubator supplemented with 5% CO_2_.

### High content analysis

Cells were cultured at a density of 2000 cells/well in 96-well plates in DMEM overnight, and then treated with GRR10W4-Alexa488 (5 μM) in serum-free DMEM for 2 h. After that, cells were washed with PBS, fixed with 4% paraformaldehyde for 15 min and stained with Alexa Fluor568-phalloidin for 30 min and DAPI for 1 min. Cells were then imaged by an automated microscope IN Cell Analyzer 2200 (GE Healthcare Life Sciences). Cell-based high content analysis of cellular uptake of GRR10W4-Alexa488 was performed using IN Cell Investigator software (GE Healthcare Life Sciences) that automatically quantified different fluorescent labels associated with pre-defined cytoplasmic and nuclear regions. Multi-parametric quantitative data sets were then converted into colour gradient-coded Heat-maps using Spotfire data visualization and analytics software (TIBCO Software Inc).

### Confocal microscopy

Peptide and doxorubicin uptake into cells was investigated by confocal microscopy. Cells were plated onto a cover slip at a density of 2.5 × 10^4^ in a 24-well plate in DMEM media with 10% FBS. After 24 h, they were incubated with either GRR10-Alexa 488 or GRR10W4-Alexa 488 in serum-free DMEM at 37 °C or 4 °C. Subsequently, cells were washed with PBS, fixed with 4% paraformaldehyde in PBS for 15 min at room temperature, and washed with PBS three times. The coverslips were then mounted on a glass slide with VECTASHIELD mounting medium containing nuclear counter stain DAPI (Vector Laboratories, Burlingame, USA). Samples were imaged with a 63×/1.25 oil objective using Zeiss 510 Confocal Microscope (Jena, Germany). The images were collected with LSM Image Browser and analysed using ImageJ Software.

### Flow cytometry

Cells were cultured in DMEM media with 10% FBS at a density of 5 × 10^5^ cells/24-well. After 24 h, they were incubated with GRR10 or GRR10W4 in serum-free media and then trypsinized and collected for centrifugation at 400 g in 5 min. Pellets were collected and washed twice with PBS. After centrifugation, cell pellets were stained with annexin V and propidium iodide using Annexin V/Dead Cell Apoptosis Kit (Thermo Fisher). They were then diluted with incubation buffer, filtered, and analyzed using Becton Dickinson FACScalibur for fluorescence emission at 530 nm and 575 nm using 488 nm excitation. Data was analyzed using Flowing software (version 2.4). Early apoptotic and late apoptotic-necrotic cells were quantified through the combined use of the fluorescence dyes PI and annexin V-FITC, as described before^19^. Through this, the cell population can be divided in four different phases, corresponding to viable (lower left quadrant), early apoptotic (upper left quadrant), primary necrotic (lower right quadrant), and late apoptotic or necrotic (upper right quadrant) cells^20^. Cell numbers in these quadrants were counted and divided by the total number of cells to give the percentage of cells in each category.

### Hemolysis assay

EDTA-blood was centrifuged at 800 g for 10 min, whereafter plasma and buffy coat were removed. The erythrocytes were washed three times and resuspended to 5% in PBS, pH 7.4. The cells were then incubated with end-over-end rotation for 1 h at 37 °C in the presence of peptides (60 or 120 μM). 2% Triton X-100 (Sigma-Aldrich, St. Louis, USA) served as positive control. The samples were then centrifuged at 800 g for 10 min. The absorbance of hemoglobin release was measured at 540 nm and is expressed as % of Triton X-100 induced hemolysis. Results given represent mean values from triplicate measurements.

### MTT assay

The MTT assay was utilized to analyse cell viability after treatment with peptides and doxorubicin. Sterile-filtered MTT (3-(4,5-dimethylthiazolyl)-2,5-diphenyl-tetrazoliumbromide; Sigma-Aldrich, St. Louis, USA) solution (5 mg/ml in PBS) was stored protected from light at −20 °C until usage. Melanoma cells (RPMI 7951 and MeWo cells) or normal cells (fibroblasts and HaCat cells) were seeded in 96 well plates at the density of 2500 cells/well for fibroblasts and 5000 cells/well for the other cells, and grown in DMEM with 1% or 10% FBS for 24 h or 48 h. After incubation, 20 μl of the MTT solution was added to each well and the plates incubated for 1 h in CO_2_ at 37 °C. The MTT- containing medium was then removed by aspiration. In the assay, MTT is modified into a dye, blue formazan, by enzymes associated to metabolic activity. The blue formazan product generated was dissolved by the addition of 100 μl of 100% DMSO per well. The plates were then gently swirled for 10 min at room temperature to dissolve the precipitate. The absorbance was measured at 550 nm, and results given represent mean values from triplicate measurements.

### Lactate dehydrogenase (LDH) assay

The LDH assay was utilized to analyse membrane permeabilising effects of peptides on melanoma cells. Cells were grown in 96 well plates at the density of 5000 cells/well in DMEM with 1% or 10% FBS. After 24 h, the medium was replaced by DMEM with 1% or 10% FBS, and the peptides investigated were added in triplicates to different wells of the plate. The LDH Cytotoxicity Assay Kit (Pierce, Thermo Fisher) was used for quantification of LDH release from the cells. Results given represent mean values from triplicate measurements, and are given as fractional LDH release compared to the positive control consisting of 1% Triton X-100 (yielding 100% LDH release).

Fibroblasts and erythrocytes were identified as suitable due to the presence of fibroblasts in skin and the abundance of erythrocytes in blood, hence covering two distinctly different biological environments. Although much more needs to be done to assess safety and efficacy of these peptides in a potential therapeutic development context, e.g., through including a wider range of melanoma cells of different origin and in different stages of progression, as well as a wider range of toxicity indicators (other types of healthy cells, but also effects on coagulation, complement, and tissue distribution), the set of two melanoma cells and two distinctly different non-malignant cells was considered sufficient for an initial proof-of-concept study of this type.

### Liposome preparation and leakage assay

Model liposomes investigated were either anionic (DOPC/DOPS 4/1-1/1 mol/mol, DOPC/GM1 4/1-1/1 mol/mol, or DOPE/DOPC/DOPI/DOPS/CL 4.8/2.8/1/1/0.4 mol/mol; ‘mitochondria’) or zwitterionic (DOPC). The choice of the former two was motivated by the up-regulation of PS and gangliosides in cancer cells^5^,^6^,^7^,^8^. DOPE/DOPC/DOPI/DOPS/CL (4.8/2.8/1/1/0.4 mol/mol) represents a model for mitochondria cell membranes^21^, whereas DOPC models non-malignant eukaryotic cell membranes^13^,^22^. DOPS (1,2-dioleoyl-*sn*-Glycero-3-phosphoserine, monosodium salt), DOPC (1,2-dioleoyl-*sn*-Glycero-3-phosphocholine), DOPE (1,2-dioleoyl-*sn*-Glycero-3-phoshoetanolamine), DOPI (1,2-dioleoyl-*sn*-Glycero-3-phosphoinositol, monosodium salt), CL (Cardiolipin; C18:1), and GM1 (Ganglioside GM1) were all from Avanti Polar Lipids (Alabaster, USA) and of >99% purity. Because of the long, symmetric, and unsaturated acyl chains of these phospholipids, membrane cohesion is good, which facilitates stable unilamellar liposomes and well-defined supported lipid bilayers, allowing detailed data on leakage and adsorption density to be obtained. Although both melanoma, fibroblasts, and HaCat cells contain also cholesterol, this was omitted in the experiments in order to more clearly demonstrate the role of electrostatics for the peptide-membrane interactions, and for cell selectivity. The lipid mixture was dissolved in chloroform, after which solvent was removed by evaporation under vacuum overnight. Subsequently, 10 mM Tris buffer, pH 7.4, was added together with 0.1 M carboxyfluorescein (CF) (Sigma, St. Louis, USA). After hydration, the lipid mixture was subjected to eight freeze-thaw cycles, consisting of freezing in liquid nitrogen and heating to 60 °C. Unilamellar liposomes of about Ø140 nm were generated by multiple extrusions (30 passages) through polycarbonate filters (pore size 100 nm) mounted in a LipoFast miniextruder (Avestin, Ottawa, Canada) at 22 °C. Untrapped CF was removed by two subsequent gel filtrations (Sephadex G-50, GE Healthcare, Uppsala, Sweden) at 22 °C, with Tris buffer as eluent. CF release from the liposomes was obtained by monitoring the emitted fluorescence at 520 nm from liposome dispersions (10 μM lipid in 10 mM Tris, pH 7.4). An absolute leakage scale was obtained by disrupting the liposomes at the end of each experiment through addition of 0.8 mM Triton X-100 (Sigma-Aldrich, St. Louis, USA). A SPEX-fluorolog 1650 0.22-m double spectrometer (SPEX Industries, Edison, USA) was used for the liposome leakage assay. Measurements were performed in triplicate at 37 °C.

### CD spectroscopy

CD spectra were measured by a Jasco J-810 Spectropolarimeter (Jasco, Easton, USA). Measurements were performed in duplicate at 37 °C in a 10 mm quartz cuvette under stirring with a peptide concentration of 10 μM. The effect on peptide secondary structure of liposomes at a lipid concentration of 100 μM was monitored in the range 200–260 nm. To account for instrumental differences between measurements, background correction was performed routinely by subtraction of spectra for buffer (with or without liposomes) from spectra of the corresponding samples in the presence of peptide.

### Ellipsometry

Peptide adsorption to supported lipid bilayers was studied *in situ* by null ellipsometry, using an Optrel Multiskop (Optrel, Kleinmachnow, Germany) equipped with a 100 mW Nd:YAG laser (JDS Uniphase, Milpitas, USA). All measurements were carried out at 532 nm and an angle of incidence of 67.66° in a 5 mL cuvette under stirring (300 rpm). Both the principles of null ellipsometry and the procedures used have been described before^23^. In brief, by monitoring the change in the state of polarization of light reflected at a surface in the absence and presence of an adsorbed layer, the mean refractive index (n) and layer thickness (d) of the adsorbed layer can be obtained. From the thickness and refractive index the adsorbed amount (Γ) was calculated according to:


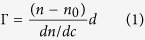


where n_0_ is the refractive index of the bulk solution (1.3347), and dn/dc the refractive index increment (0.154 cm^3^/g). Corrections were routinely done for changes in bulk refractive index caused by changes in temperature and excess electrolyte concentration.

The zwitterionic phospholipid bilayers were deposited on silica surfaces by co-adsorption from a mixed micellar solution containing DOPC and n-dodecyl-β-D-maltoside (DDM; ≥98% purity, Sigma-Aldrich, St. Louis, USA), as described in detail previously^24^. In brief, the mixed micellar solution was formed by addition of 19 mM DDM in water to DOPC dry lipid films, followed by stirring over night, yielding a solution containing 97.3 mol% DDM. This micellar solution was added to the cuvette at 25 °C, and the following adsorption monitored as a function of time. When adsorption had stabilised, rinsing with Milli-Q water at 5 ml/min was initiated to remove mixed micelles from solution and surfactant from the substrate. By repeating this procedure and subsequently lowering the concentration of the micellar solution, stable and densely packed bilayers are formed, with structural characteristics similar to those of bulk lamellar structures of the lipids^24^.

As sub-bilayer and patchy adsorption resulted from the above mixed micelle approach in the case of the anionic lipid mixtures, supported lipid bilayers were generated from liposome adsorption for these. DOPC/DOPS (4/1-1/1 mol/mol), DOPC/GM1 (4/1-1/1 mol/mol), and ‘mitochondria’ (DOPE/DOPC/DOPI/DOPS/CL 4.8/2.8/1/1/0.4 mol/mol) liposomes were prepared as described above, but the dried lipid films re-suspended in Tris buffer only with no CF present. In order to avoid adsorption of peptide directly at the silica substrate (surface potential −40 mV, contact angle <10°)^25^ through any defects of the supported lipid layer, poly-L-lysine (M_w_ = 170 kDa, Sigma-Aldrich, St. Louis, USA) was preadsorbed from water prior to lipid addition to an amount of 0.045 ± 0.01 mg/m^2^, followed by removal of non-adsorbed poly-L-lysine by rinsing with water at 5 mL/min for 20 minutes. Water in the cuvette was then replaced by 10 mM Tris, pH 7.4, containing also 150 mM NaCl, followed by addition of liposomes in buffer at a lipid concentration of 20 μM, and subsequently by rinsing with buffer (5 mL/min for 15 minutes) when the liposome adsorption had stabilised. The final layers formed were characterized by an area/molecule of 60 ± 10 Å^2^ in all cases, suggesting that layers fairly close to complete bilayers are formed for these lipids. (It should here be noted, that pLys pre-adsorbed (0.045 ± 0.01 mg/m^2^) was ≈100-fold lower than lipid adsorption corresponding to bilayer formation (≈4.5 mg/m^2^). Moreover, due to the exceedingly flat conformation of pre-adsorbed pLys (thickness ≈1–2 Å, train fraction close to 100%), desorption rates are extremely long or in essence irreversible^26^. In comparison, bilayer formation is very fast (minutes), hence giving no time for the minute amount of pLys to be desorbed. The absence of exchange/extraction of pre-adsorbed pLys is indicated also by the lack of transient maxima in the lipid adsorption curves, as well as by the incomplete bilayer formation in the absence of pLys). After lipid bilayer formation, the cuvette content was replaced by 10 mM Tris buffer at a rate of 5 mL/min over a period of 30 minutes. After stabilization for 40 minutes, peptide was added to a concentration of 0.01 μM, followed by three subsequent peptide additions to 0.1 μM, 0.5 μM, and 1 μM, in all cases monitoring the adsorption for one hour. All measurements were made in at least duplicate at 25 °C.

Ellipsometry has been previously demonstrated to provide peptide binding information equivalent to that obtained from isothermal calorimetry^27^ and fluorescence spectroscopy^28^.

### Size measurements

Liposome size determination in the absence and presence of peptide (at 1 μM in 10 mM Tris, pH 7.4) was investigated by nanoparticle tracking analysis, using a NanoSight NS500 (NanoSight Ltd., Amesbury, UK), equipped with a 75 mW laser at 532 nm, and the NTA 2.3 analytical software. Within an illumination device mounted under a microscope, particles passing through the beam path were visualized as small dots. From the displacements measured, diffusion coefficients of individual particles were obtained, the size of which was subsequently obtained from the Stokes-Einstein equation. The lipid concentration used for sample preparation was 10 μM, and measurements performed at 22 °C, 1 hr after sample preparation. Due to low particle concentration necessary for NTA (10^8^–10^9^ particles/mL), samples were diluted with 10 mM Tris, pH 7.4 just prior to measurement. Each size measurement was based on a 30 s video, analyzed using a screen gain of 10, blur: auto, and a detection threshold of 8–10. Data are presented as the average and standard deviation of the three video recordings.

### Liposome zeta potential

The z-potential of liposomes in the absence and presence of peptide was determined by dynamic light scattering at a scattering angle of 173°, using a Zetasizer Nano ZS (Malvern Instruments, Malvern, UK) and calculated according to the Smoluchowski model. The lipid concentration was fixed at 50 μM, while peptide concentrations was varied between 0 and 2 μM in 10 mM Tris, pH 7.4. Peptide/liposome mixtures were incubated for one hour before measurements were initiated, in each case monitoring size over time to ensure absence of time-dependent effects. Measurements were performed in duplicate at 25 °C.

### Doxorubicin uptake

RPMI 7951 or MeWo cells were treated with 0.1 μM Doxorubicin with or without 5 μM GRR10-Alexa488 or 5 μM GRR10W4-Alexa488 for 2 h, whereafter peptide (green) and doxorubicin (red) uptake was monitored by confocal microscopy. Toxicity after co-treatment with doxorubicin and GRR10 or GRR10W4 peptides on RPMI 7951 and MeWo cells was determined by MTT assay (described above) of cells treated with 0.1 μM doxorubicin and either 5 μM GRR10 or 5 μM GRR10W4 for 24 h.

### Particle uptake

Nanoparticles were coated with either GRR10 or GRR10W4 peptides (2 μM peptide/100 ppm particle) in Tris pH 7.4 buffer for 3 h. RPMI 7951 cells were then incubated with the peptide-coated nanoparticles (equivalent to 5, 25 or 50 nM peptides) for 2 h and particle uptake monitored by confocal microscopy.

### Statistics

Values are reported as means ± standard deviation of the means. To determine significance, analysis of variance with ANOVA was used as indicated in the figure legends, where “n” denotes number of independent experiments. Significance was accepted at p < 0.05.

## Results

### Peptide binding

**Figure 1:**
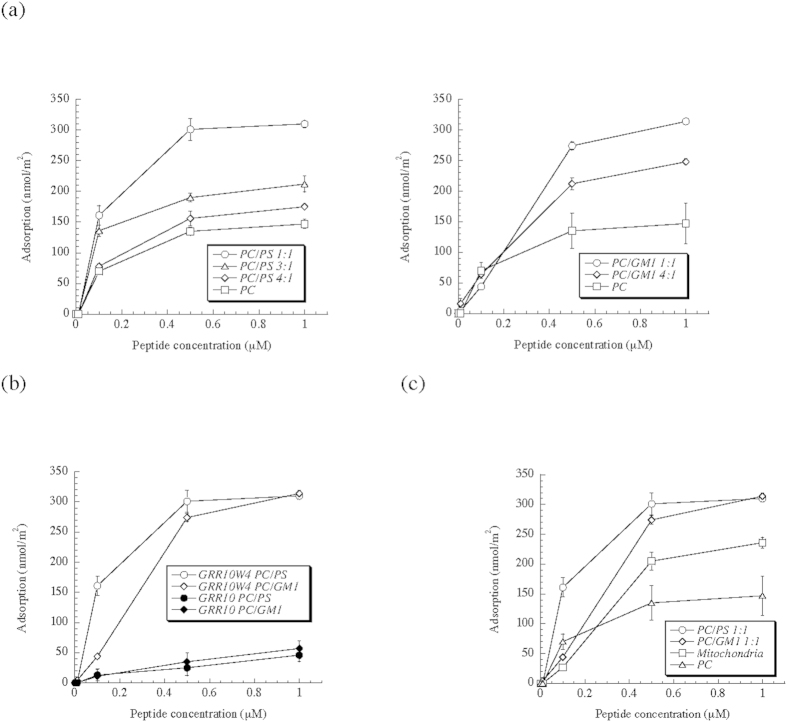
Ellipsometry results on the adsorption of GRR10 and GRR10W4N at supported lipid membranes. (**a**) Effect of negatively charged DOPS and ganglioside GM1 content on the adsorption of GRR10W4N to DOPC/DOPS (left) and DOPC/GM1 (right) membranes. (**b**) Comparison of GRR10 and GRR10W4 adsorption to DOPC/DOPS and DOPC/GM1 membranes (**c**) Comparison of GRR10W4 adsorption to different negatively charged membranes of relevance to cancer cell uptake, as well as zwitterionic DOPC membranes. *p < 0.05 for PS and GM1 effect (**a**), GRR10W vs GRR10 (**b**), as well as PC/PS and PS/GM1 vs ‘mitochondia’ and PC (**c**).

**Figure 2:**
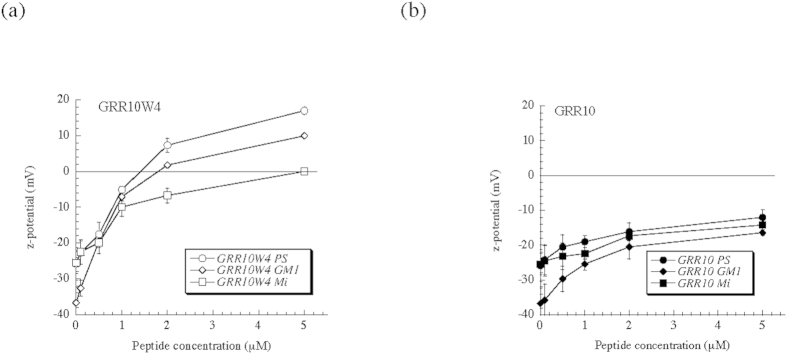
z-potential of DOPC/DOPS (3/1 mol/mol), DOPC/GM1 (3/1 mol/mol), and ‘mitochondria’ (Mi), liposomes in the presence of GRR10W4 (**a**) and GRR10 (**b**) at the indicated concentrations. The point of zero potential is included to guide the eye. *p < 0.05 for PC/PS vs PC/GM1 vs ‘mitochondia’, as well as for GRR10W vs GRR10.

**Figure 3:**
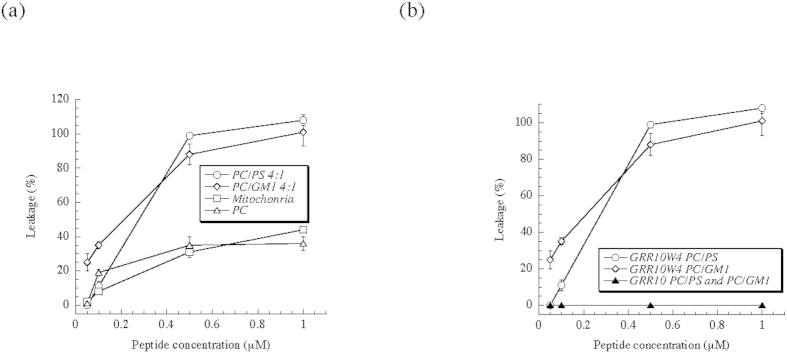
Peptide-induced liposome leakage for negatively charged (DOPC/DOPS, DOPC/GM1, ‘mitochondria’) membranes, as well as zwitterionic DOPC membranes. *p < 0.05 for PC/PS and PC/GM1 vs ‘mitochondia’ and PC, as well as for GRR10W vs GRR10.

A key feature of the membrane composition of cancer cells is the relative abundance of anionic (phospho)lipids, including phosphatidylserine and gangliosides in the outer membrane^5^,^6^,^7^,^8^. In order to monitor the effects of such anionic lipids on membrane interactions, peptide binding to supported lipid bilayers containing these was first investigated. Ellipsometry results in Fig. 1a show that adsorption of GRR10W4 increases with the anionic lipid content for both PS and GM1. Comparing the latter two, it can be noted that the saturation adsorption is comparable, in agreement with the relatively similar electrostatic potential of these surfaces. Saturation adsorption in both these cases is about 300 nmol/m^2^, corresponding to about 1 peptide per 11 lipid molecules in the outer membrane leaflet. Demonstrating the importance of the hydrophobic W-tag, Fig. 1b shows the substantially lower adsorption of GRR10 than of GRR10W4 at the PS- and GM1-containing membranes. Finally, Fig. 1c shows the adsorption selectivity of GRR10W4, with high adsorption at PS- and GM1-rich membranes, intermediate binding at mitochondria-mimicking membranes, and much lower peptide adsorption to zwitterionic DOPC membrane. Similarly, z-potential measurements show that the z-potential of the three anionic liposomes investigated became substantially less negative, or even reversing to a net positive charge, on addition of GRR10W, while the effect of GRR10 is substantially smaller (Fig. 2), reflecting the lower adsorption of this peptide to these lipid membranes (Fig. 1). Furthermore, the ‘mitochondria’ liposome only reaches charge neutrality at the highest peptide concentration investigated, whereas both DOPC/DOPS and DOPC/GM1 reverse their charge at the same conditions. The latter finding reflects the lower adsorption of GRR10W at ‘mitochondria’ than at DOPC/DOPS and DOPC/GM1 membranes (Fig. 1) and correlates also to the higher leakage induction of this peptide in DOPC/DOPS and DOPC/GM1 liposomes (Fig. 3). Together, these results show GRR10W4 to display pronounced adsorption selectivity, largely, but not completely, driven by electrostatic interactions.

### Membrane destabilization

Next, the consequences of peptide binding selectivity for membrane stability were investigated. Liposome leakage experiments in Fig. 3a show that liposomes containing PS or GM1 are potently destabilized by GRR10W4. Furthermore, Fig. S1 shows that liposome size is the same in the absence and presence of GRR10W4. Thus, there is no peptide-induced liposome flocculation, and the leakage results can be straightforwardly interpreted as being due to peptide-induced defect formation in the liposome membrane. Also, CD spectroscopy data, although being complex due to side-group contributions, shows the conformation of GRR10W4 to be unaffected by its membrane binding (Fig. S2), hence no peptide conformational effects complicate the interpretation of the leakage results. Mirroring the adsorption, peptide-induced liposome destabilization follows the order PS≈GM1> ‘mitochondria’, whereas zwitterionic DOPC liposomes are least destabilized by the peptide. Also mirroring peptide adsorption, peptide-induced liposome lysis is much lower for GRR10 than for GRR10W4, again demonstrating the effect of the W-tag for peptide activity. Together, these results show a direct correlation between peptide adsorption selectivity and membrane lysis selectivity.

### Cell uptake

**Figure 4:**
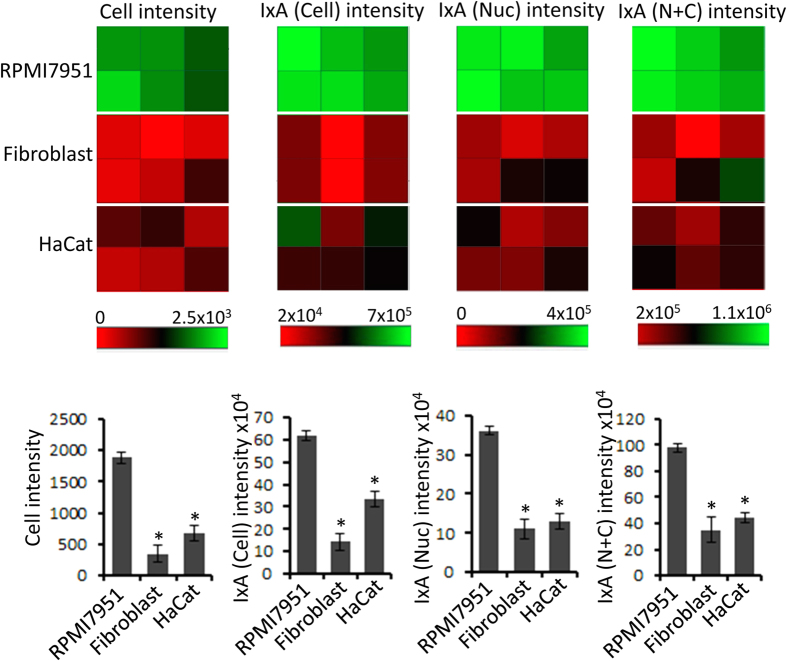
Selective uptake of GRR10W4 in melanoma cells. Fibroblast, HaCat and RPMI 7951 cells were cultured overnight, and then incubated with GRR10W4-Alexa488 (5 μM) for 2 h. After that cells were washed, fixed and stained with Alexa Fluor568-phalloidin and DAPI before being subjected to high content analysis. (**a**) Heat map for normalized fluorescence intensity representing GRR10W4-Alexa488 uptake in RPMI 7951 cells, HaCat cells and fibroblasts. (**b**) Comparison of GRR10W4-Alexa488 uptake in melanoma and non-malignant cells. *Cell intensity*, average intensity of pixels within the cells; *IxA (Cell) intensity*, nucleus average intensity multiplied by nuclear area; *IxA (Cell) intensity*, cytoplasm average intensity multiplied by cytoplasmic area; *IxA* (*N* + *C*) *intensity*, cell average intensity multiplied by cellular area. *p < 0.05 compared to RPMI 7951 cells.

**Figure 5:**
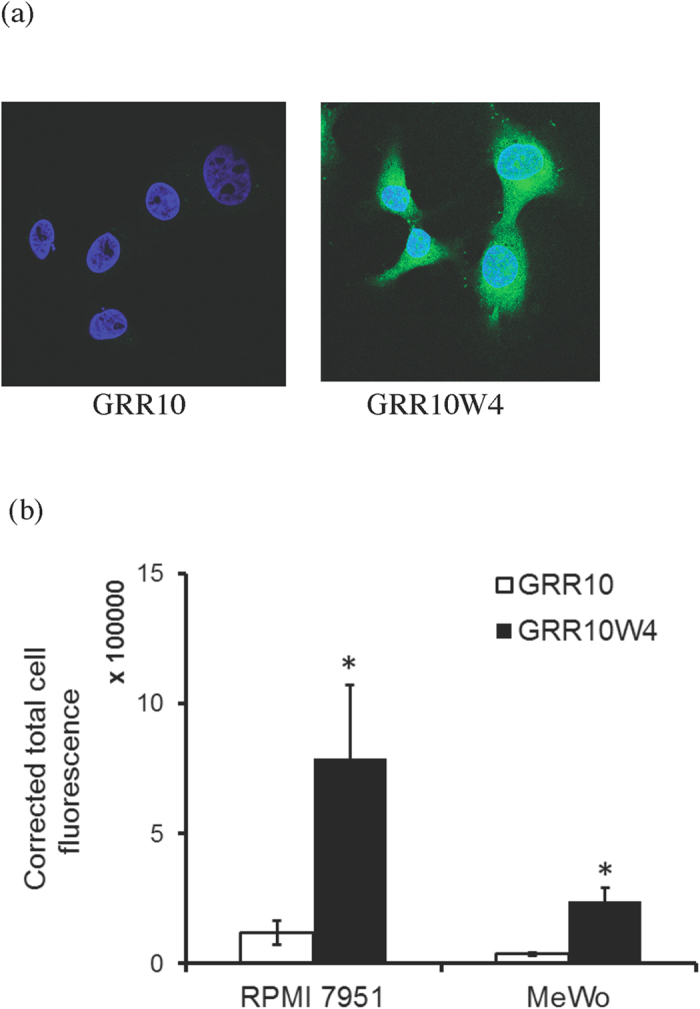
(**a**) Representative confocal microscopy images of GRR10 and GRR10W4 uptake in RPMI7951 melanoma cells. (**b**) Quantification of peptide uptake. For (**a**), cells were incubated with 10 μM Alexa488-conjugated peptide for 2 h, whereas in (**b**), the peptide concentration used was 5 μM for 2 h. *p < 0.05 compared to GRR10.

**Figure 6:**
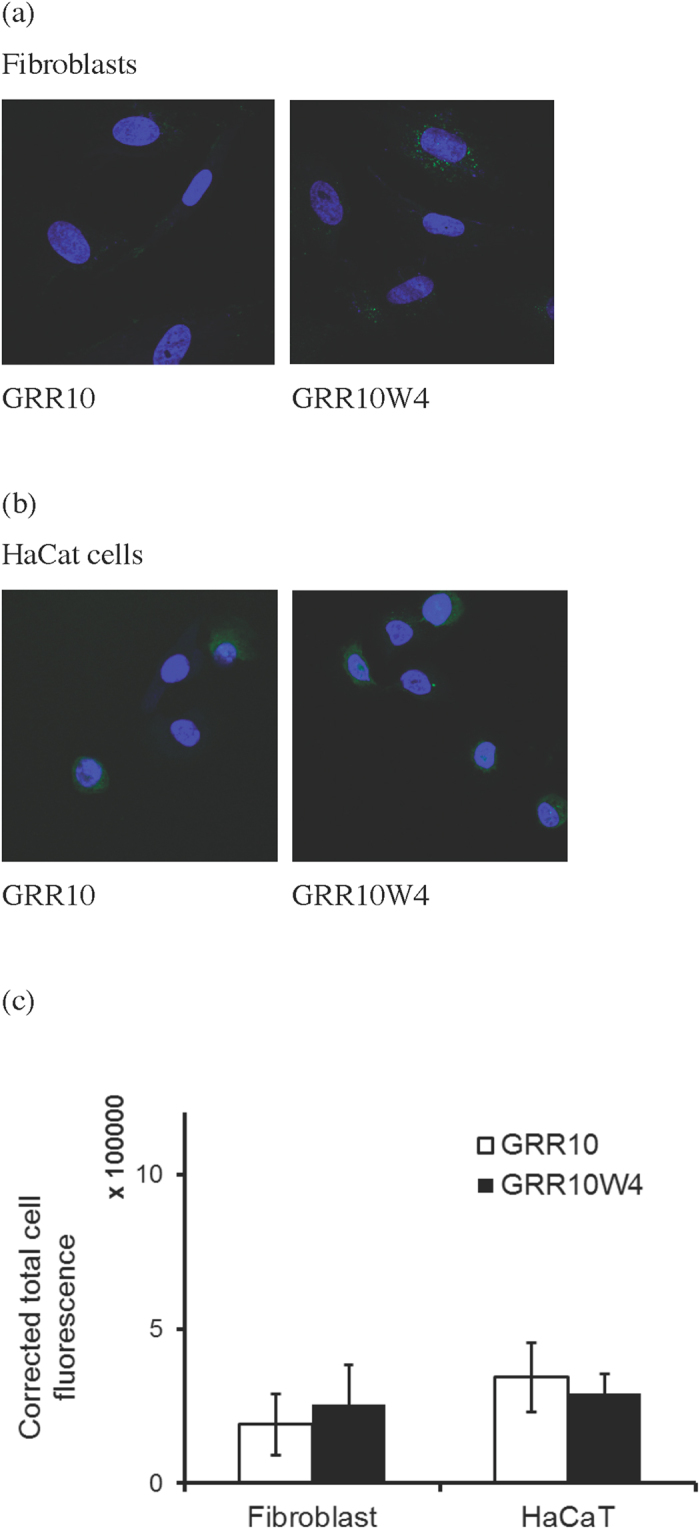
Peptide uptake in fibroblasts and HaCat keratinocytes. Shown are both representative confocal microscopy images (**a,b**) and quantification of peptide uptake (**c**) after exposure at 5 μM for 2 h. No significant effect was observed between GRR10 and GRR10W4.

Having demonstrated selectivity in the model lipid membranes, we next investigated internalization of GRR10 and GRR10W4 in RPMI 7951 and MeWo melanoma cells. In agreement with the model lipid membrane investigations, GRR10 is hardly internalized at all in these cells, whereas GRR10W4 is incorporated selectively in melanoma cells (Figs 4 and 5), a process occurring over relatively long time (Fig. S3). Quantitatively, peptide uptake is higher in RPMI 7951 than in MeWo cells, indicating differences between these cells in membrane composition as well as other aspects, not captured in the model membrane systems. Peptide internalization in both RPMI 7951 and MeWo melanoma cells is strongly suppressed at 4 ^o^C (Fig. S4), which may be compatible either with suppressed endocytosis or with decreased translocation, caused by reduced membrane fluidity, at the lower temperature^29^. In contrast to melanoma cells, and in agreement with the model lipid membrane results, non-malignant fibroblasts and keratinocyte cells display substantially lower cell uptake, not affected by the presence of the W-tag in GRR10W4 (Figs 4 and 6). Taken together, these results demonstrate a good selectivity in peptide uptake between melanoma and non-malignant cells, as well as a good correlation between peptide-induced membrane destabilization in the model lipid systems, on one hand, and cellular uptake, on the other.

### Cell toxicity

**Figure 7:**
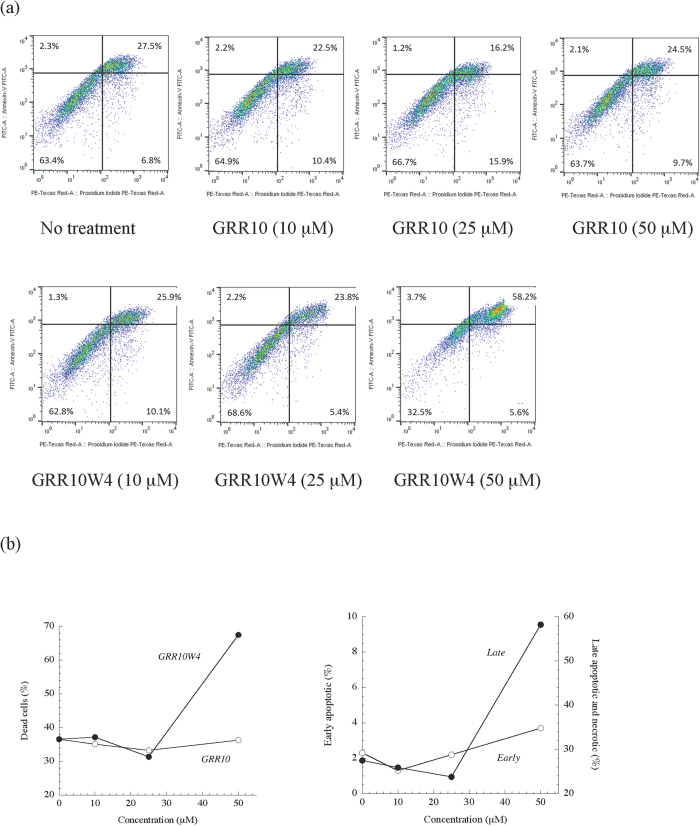
(**a**) Flow cytometry results on peptide toxicity against RPMI 7951 melanoma. Cells were incubated with peptides at the indicated concentrations for 48 h, followed by staining with annexin-V and PI and flow cytometric analysis. (**b**) Quantification of the fraction of dead cells after peptide exposure (left), as well as of early apoptotic and late apoptotic/necrotic cells (right).

**Figure 8:**
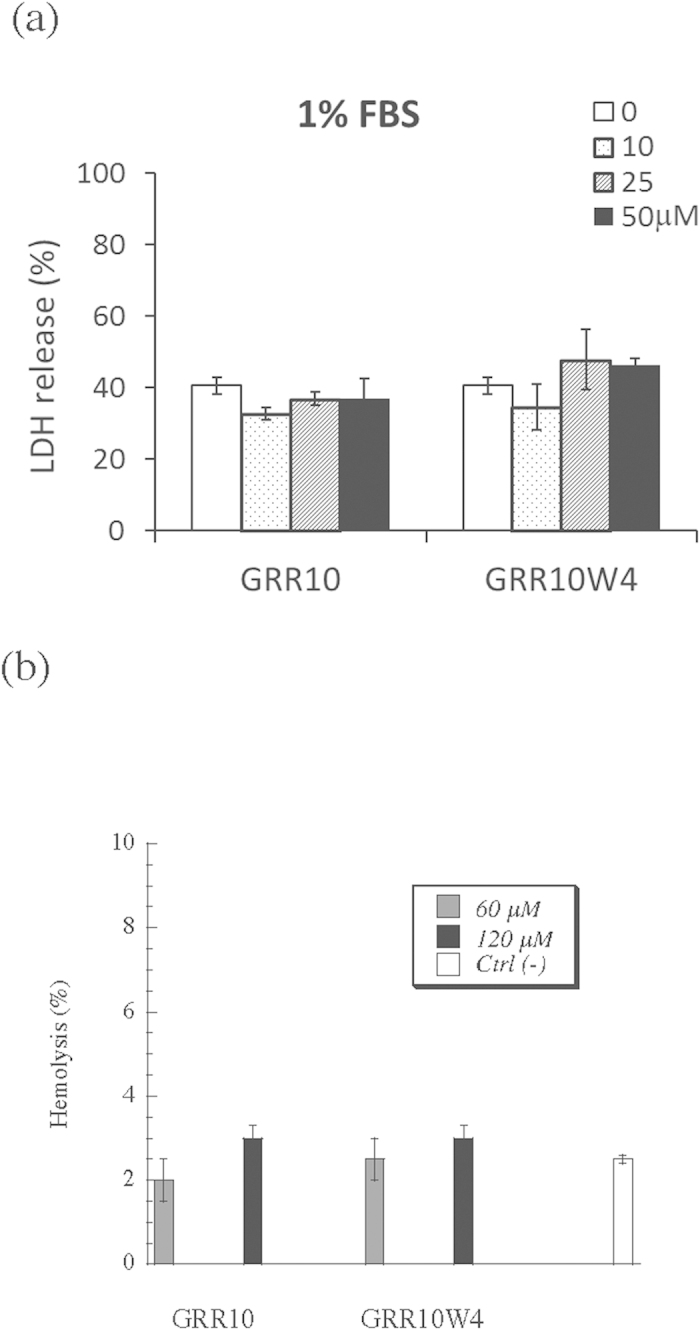
Toxicity of GRR10 and GRR10W4 on non-malignant cells. (**a**) Fibroblasts were exposed to the peptides at the indicated concentrations for 48 h in 1% FBS, after which cell toxicity was monitored by LDH assay. As seen, the indicated peptides display no significant toxicity up to at least 50 μM. Analogous results on low toxicity was found for erythrocytes in hemolysis assay, with no significant peptide effect up to 120 μM (**b**).

Next, we investigated the consequences of peptide uptake in melanoma for their cell toxicity. GRR10W4 but not GRR10 results in a dose-dependent increase in the fraction of dead cells for the melanoma cell line RPMI 7951 (Fig. 7). Quantitatively, the fraction of dead and apoptotic RPMI 7951 cells increases strongly at a GRR10W4 concentration of 50 μM, whereas it remains unaffected up to 50 μM of GRR10, i.e., in the absence of the membrane-anchoring W-tag (Fig. 7). As shown in Fig. S5, similar effects were found for MeWo melanoma cells. While both the fraction of early apoptotic cells and that of late apoptotic/necrotic cells increase in the presence of GRR10W4 for both RPMI 7951 and MeWo cells, the dominating effect is seen for the late apoptotic and necrotic cells. In analogy to the flow cytometry results, MTT assay showed GRR10W4, but not GRR10, to be toxic against melanoma cells at the peptide concentrations investigated (Fig. S6). In contrast to melanoma cells, toxicity of both GRR10 and GRR10W4 was low against non-malignant cells, demonstrated for both fibroblasts and erythrocytes, showing these peptides not to induce any membrane-permeabilizing effects beyond the background leakage in the case of fibroblasts, up to a peptide concentrations of at least 50 μM. Analogously, hemolysis of erythrocytes was very low, at the background level of the negative control, up to a peptide concentration of at least 120 μM (Fig. 8). Therefore, the results show that there is a good correlation between cell internalization and peptide-induced toxicity.

### Peptide facilitation of doxorubicin and nanoparticle uptake

**Figure 9:**
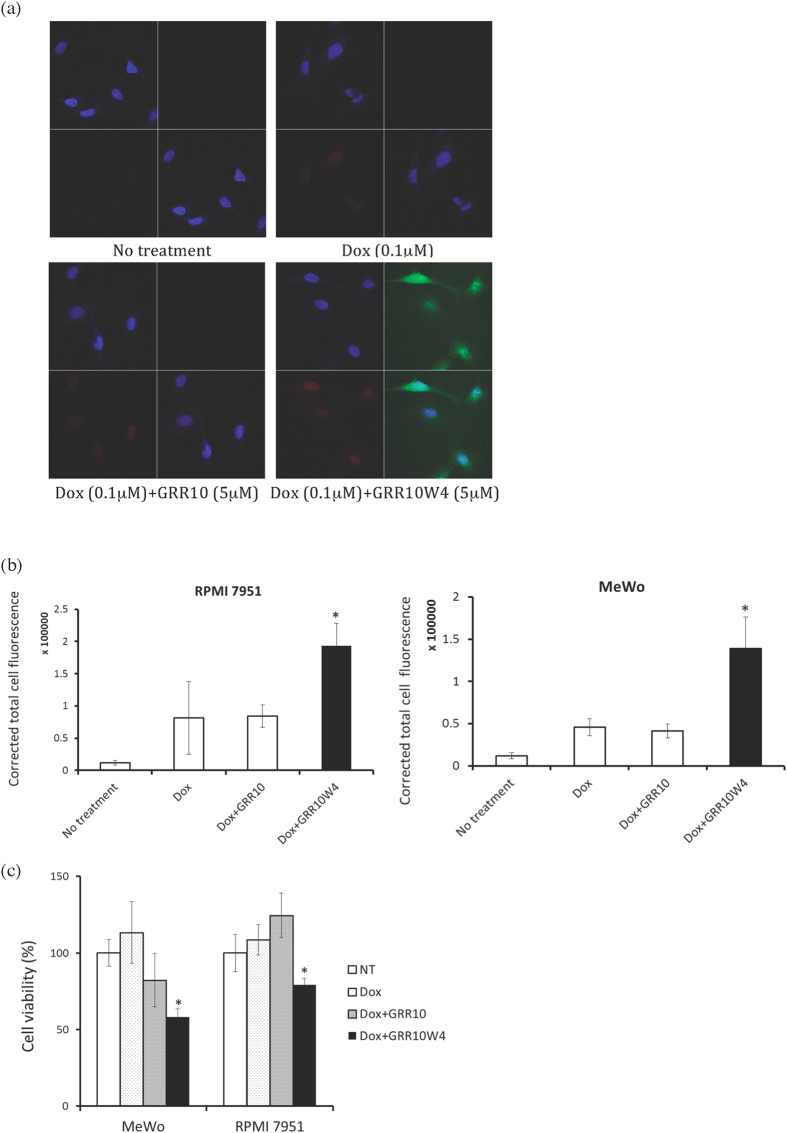
Enhanced uptake and toxicity of doxorubicin by GRR10W4 in melanoma cells. (**a**) Representative confocal microscopy images of peptide and doxorubicin uptake in RPMI 7951 melanoma cells and (**b**) quantification of doxorubicin uptake after co-treatment with doxorubicin and peptides in RPMI 7951 and MeWo cells. For both (**a**,**b**), RPMI 7951 cells or MeWo cells were treated with 0.1 μM doxorubicin and 5 μM GRR10-Alexa488 or 5 μM GRR10W4-Alexa488 for 2 h and subjected to confocal microscopy. (**c**) Toxicity of co-treatment with doxorubicin and GRR10 or GRR10W4 peptides on RPMI 7951 and MeWo cells. Cells were treated with 0.1 μM doxorubicin and 5 μM GRR10 or 5 μM GRR10W4 for 24 h and subjected to cell viability assay by MTT. *p < 0.05 compared to no treatment group.

**Figure 10:**
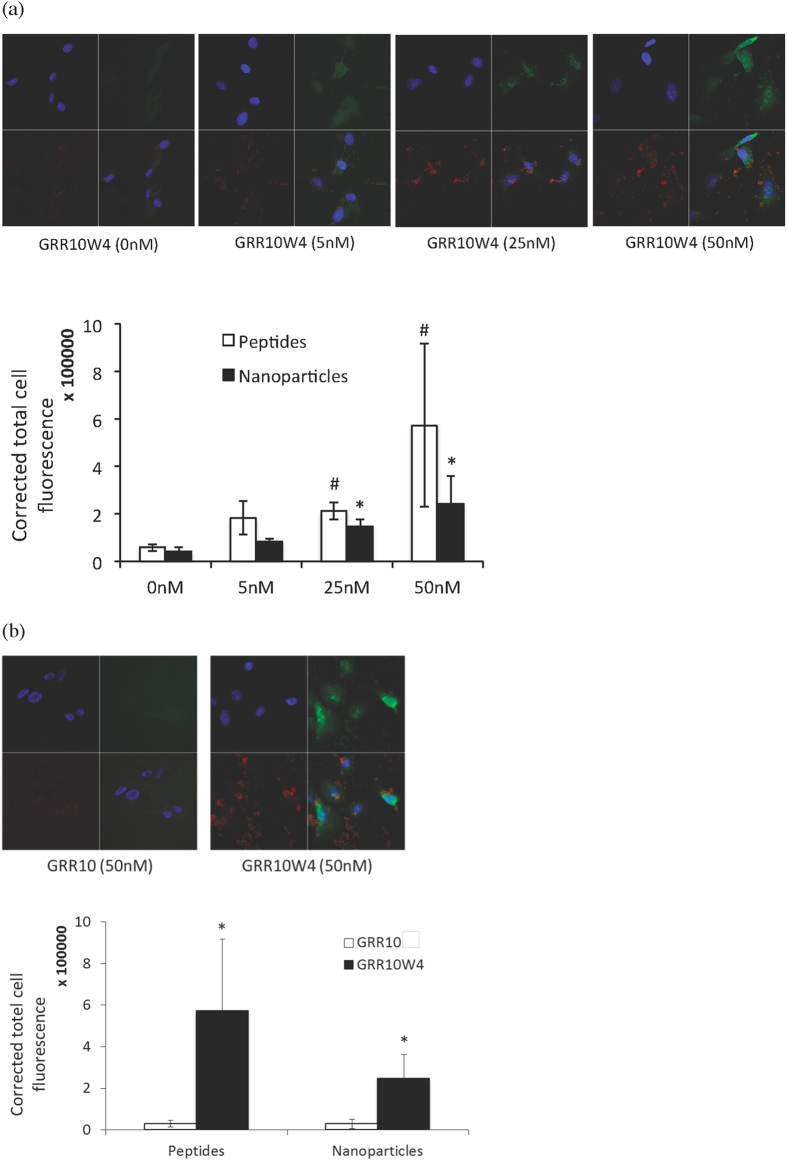
Enhanced uptake of nanoparticles by GRR10W4 in melanoma cells. Nanoparticles were coated with either GRR10 or GRR10W4 peptides in Tris buffer (2 μM peptide/100 ppm particles) for 3 h. RPMI 7951 cells were then incubated with the peptide-coated nanoparticles for 2 h and subjected to confocal microscopy. (**a**) Representative confocal microscopy images of GRR10W4 peptide (green) and nanoparticle (red) uptake in RPMI 7951 melanoma cells (upper) and quantification of GRR10W4 peptide and nanoparticle uptake (lower). ^#,^*p < 0.05 compared to no treatment (0 nM) of the same group. (**b**) Comparison of the effects of GRR10 (50 nM) and GRR10W4 (50 nM) peptides on nanoparticle uptake (upper) and corresponding quantification (lower). *p < 0.05 compared to GRR10.

Apart from direct therapeutic opportunities offered by the selective cytotoxicity of GRR10W4 against melanoma, one can envision several other contexts, where selective peptide uptake in melanoma and other types of cancer cells can be employed to obtain functional advantages. For example, one could imagine combining cancer-selective peptides with low Mw anticancer drugs, where the outer membrane defect formation due to the peptide may facilitate uptake of the latter and result in increased anticancer activity due to increased cell internalization. In order to investigate this, doxorubicin uptake into melanoma cells was monitored in the absence and presence of peptide. Confocal microscopy experiments in Fig. 9 show that GRR10W4, but not GRR10 facilitates cell uptake of doxorubicin into both RPMI 7952 and MeWo melanoma, resulting in enhanced cell toxicity. Analogously, peptide-mediated cell internalization effects are so potent that GRR10W4 can be used also as a surface coating for nanoparticles to obtain not only targeting to melanoma cells, but also facilitated nanoparticle uptake by these cells. Demonstrating this, model fluorescent polymer nanoparticles were incubated with GRR10W4 and GRR10 and cell internalization subsequently monitored. Using this approach, an increased particle uptake in melanoma was indeed observed for GRR10W4, but not for GRR10 (Fig. 10).

## Discussion

Although much remains to be elucidated regarding the mode-of-action of cationic and amphiphilic peptides as anticancer agents, these peptides are thought to work by inducing either necrosis or apoptosis, or both^6^. For example, lactoferricin displays anticancer effects by inducing apoptosis in human cancer cells, including human breast carcinoma cells and monocytic leukemia cells^30^,^31^,^32^. Analogously, SALF was demonstrated to be efficiently suppressive in a tumor xenograft mouse model, an effect caused by peptide-induced apoptosis through the death receptor/NF-κB pathway^33^. Necrosis, in turn, involves degeneration of the cytoplasm and the mitochondrial matrix, extracellular release of cytoplasmic content, and chromatin flocculation^34^. Examples of peptides displaying such effects include Kahalalide F^35^ and BF-30 36. Yet other (potential) anticancer mechanisms of cationic amphiphilic peptides include interference with functional proteins, as well as immunomodulary effects^6^.

The specificity of cationic amphiphilic peptides to cancer cells through these mechanisms seems to originate from differences in membrane composition between cancer cells and non-malignant ones, e.g., related to an effective up-regulation of PS and sialic acid-containing compounds (e.g., gangliosides), while non-cancer cells exhibits a close to neutral charge due to zwitterionic phosphatidylcholine and sphingomyelin^6^,^7^,^8^. Mirroring these previous observations, the present investigations demonstrate that both peptide binding density to lipid membranes and resulting membrane destabilization depend sentitively on the presence of both phosphatidylserine and gangliosides, thus providing mechanistic support for peptide selectivity. Once cationic and amphiphilic peptides have reached the cytosol, they may also interact strongly with negatively charged components of the mitochondrial membrane, notably cardiolipin. In addition, mitochondrial membranes of different melanoma have been demonstrated to be substantially enriched also in PS^37^. Through the high content of anionic lipid components such as PS and cardiolipin, cationic amphiphilic peptides may bind to, and destabilize, mitochondria membranes, as clearly demonstrated in the present study by the combination of ellipsometry, z-potential, and liposome leakage measurements for mitochondria-mimicking membranes. Destabilization of mitochondria membrane may, in turn, result in the release of cytochrome c and subsequent apoptosis induction^38^,^39^.

Although not addressing biophysical mechanisms in any greater detail, the influence of anionic lipid components for membrane interactions and anticancer effects of cationic amphiphilic peptides has been reported previously. Thus, Iwasaki *et al.* investigated the effects of PS density at cancer cell surfaces and found this to correlate to the sensitivity of these cells to exposure to peptides derived from beetle defensins^40^. Similarly, Gaspar *et al.* demonstrated that electrostatic interactions play a key role for composition-dependent driven binding of SVS-1 and its subsequent cell effects^41^, as did Riedl *et al.* for lactoferricin^42^. For the same peptide, Sinthuvanich *et al.* reported pronounced influence of membrane composition on peptide conformational change, which in turn related to activity against KB (epidermal), MDA-MB-436 (breast), MCF-7 (breast), and A549 (lung) carcinoma cells, but low toxicity against non-malignant cells such as erythrocytes and HUVEC cells^43^. In an attempt to address the origin of such effects, Risso *et al.* investigated the effects of sialyation-dependent membrane composition, and found the BMAP-27 and BMAP-28 cathelicidins to be less active against cancer cells after cleaving off the surface sialic acid residues of these cells^44^. Similarly, Miyazaki *et al.* found F5W-magainin 2 to co-localize with gangliosides on HeLa cells and to bind to gangliosides in model membranes with an affinity increasing with the number of sialic acid residues. Importantly, however, electrostatics is not the sole determinant for these interactions, as pronounced differences were observed between binding to GM1 and phosphatidylglycerol, the latter inferred to be due to differences in peptide binding location and membrane-induced peptide clustering^45^. Interestingly, the effects of membrane sugar residues in this system are opposite from those found previously for mellitin and phosphatidylinositol-containing liposomes, indicating that they depend sensitively on system^28^. Nevertheless, both studies demonstrate the importance of also carbohydrate-related steric interactions for peptide binding and membrane destabilization.

Various approaches have been employed to increase the anticancer effects of cationic amphiphilic peptides. One of these is to conjugate the peptide to another (cell-penetrating) peptide able to promote cell internalization of the first peptide. Using this approach, Liu *et al.* investigated the possibility to increase the relatively modest anticancer activity of magainin II by conjugating it to penetratin, and found the fusion peptide to be more potent than unconjugated magainin in killing cells^46^. On the other hand, such cell-penetrating peptides may display anticancer activity by themselves, as demonstrated by Delaroche *et al.*^47^ and Jobin *et al.*^48^ for the penetratin variant RW16. Hence, fusion peptides will require quantitative superior effects in order to motivate the higher complexity compared to simpler peptide optimization or modification strategies.

Besides electrostatics, hydrophobic interactions play an important role for membrane binding and destabilization by anticancer peptides. For RW16 (RRWRRWWRRWWRRWRR), tryptophan insertion was speculated to be of particular importance for its cell activity, notably suppression of proliferation and mobility of EF cancer cells at retained low toxicity against non-malignant cells^48^. Considering the abundance of R and W residues in RW16, these findings, as well as the demonstrated perturbation of zwitterionic and anionic lipid membranes, are in good agreement with our findings for GRR10W4 (GRRPRPRPRPWWWW). In addition, as clearly demonstrated in the present investigation, end-modification with an oligomeric W tags allows cancer cell uptake and toxicity to be further increased for R-rich peptides. Somewhat analogously, Huang *et al.* found increased anticancer activity against human cervix cells of V13K after increasing its hydrophobicity through A- > L substitutions^49^, while Chu *et al.* reported boosted anticancer activity of short cationic amphiphilic peptides after addition of hydrophobic β-naphthylalanine residues to their termini^50^. In contrast to approaches involving either aliphatic conjugations/modifications or an increased mean hydrophobicity, however, end-tagging with aromatic amino acid stretches provides opportunities for high, but *selective*, membrane activity^13^. For bulky groups such as W and F, which require substantial area expansion, insertion occurs only when electrostatics is sufficient to overcome the free energy penalty associated with lateral membrane expansion^16^. As demonstrated previously, W/F-tagging offers opportunities for selective antimicrobial peptides, effectively lysing negatively charged (bacteria) membranes and allowing potency and salt resistance to be maintained down to 4–7 amino acids in the hydrophilic template peptide, yet displaying very limited lysis of zwitterionic membranes and eukaryotic cell toxicity^14^. In fact, the selectivity of these peptides allows bacteria to be efficiently killed in blood, at simultaneously negligible hemolysis^16^. It also allows efficient activity against ‘superbugs’, including methicillin-resistant *S. aureus* (MRSA), vancomycin-resistant enterococci, and multi-drug resistant *P. aeruginosa*, at very limited cell toxicity^15^. Clearly, the results of the present investigation show this selectivity to extend also to one between melanoma cells and non-malignant cells.

Due to membrane-destabilizing effects, anticancer peptides may facilitate cell uptake also of Mw compounds and result in additive or even synergistic effects. For example, doxorubicin displays broad-spectrum antitumor activity. However, since it is taken up by tumor cells through passive diffusion, the overall response rate of doxorubicin is only about 30%^51^,^52^. Hence, the combination or conjugation of doxorubicin with membrane-active anticancer peptides may facilitate internalization of doxorubicin, and translate into increased anticancer activity, as demonstrated in the present investigation for GRR10W4 and melanoma. Similarly, Zhao *et al.* investigated the combination of doxorubicin, as well as its isomer epirubicin, with the peptides HPRP-A1 and HPRP-A2. It was found that the combination of doxorubicin/epirubicin with the membrane-active peptides increased the *in vitro* activity of these drugs against different cancer cells, as well as their activity in a HeLa xenograft model in BALB/c nude mice^53^. Similar effects were found for cecropin in combination with S-fluorouracil and cytarabine^54^ and for OLP-1 in combination with doxorubicin^55^. Along the same line, Sugahara investigated the peptide iRGD either conjugated to, or co-administered with, different types of cancer drugs, and found synergistic effects in both cases, but with quantitatively slightly more pronounced effects on co-administration^56^. Such peptide-induced ‘opening up’ of the cancer cell membrane offers opportunities not only when cell internalization is poor, but also for multi-drug resistant cells characterized by an up-regulated efflux machinery^6^.

As demonstrated in the present investigation, GRR10W4 is able to facilitate not only uptake of low-Mw drugs such as doxorubicin, but also selective (targeted) uptake of nanoparticulate drug delivery systems, here modelled by carboxylated polyacrylonitrile-based nanoparticles. Similarly, Liu *et al.* investigated poly(lactic-co-glycolic acid) nanoparticles, modified by either L-R8 or D-R8 through a maleimide-mediated covalent conjugating procedure, as a carrier for oral delivery of insulin. Using a Caco-2 cell monolayer model, R8-modified nanoparticles were found to display increased cellular uptake^57^. Similar positive effects of arginine-rich peptides on polymer nanoparticles were reported by Almeida *et al.*, who reported on peptide-conjugated poly[(9,9-di-n-octylfluorenyl-2,7-diyl)-alt (benzo[1, 2, 3]thiadiazol-4,8-diyl)] nanoparticles, and found TAT-conjugated particles to display the highest particle uptake into HeLa cells^58^. Positive effects of TAT-conjugation were found also by Hossein *et al.* for Au/PEG hybrid nanoparticles^59^ and by Welsher at al. for polystyrene/quantum dot-based fluorescent nanoparticles modified with TAT peptide^60^. Apart from the peptide sequence, however, also the accessibility for binding affects cell internalization of peptide-conjugated nanoparticles. Thus, Jeong *et al.* investigated how the spacer arm length in cell-penetrating peptides influences chitosan/siRNA nanoparticle delivery for pulmonary inflammation treatment, by using an R9 with a varying number of glycine units between the peptide and the nanoparticle. In doing so, these authors found R9G10-chitosan to be much more effective in intracellular delivery of genes compared with both non-modified chitosan (without the peptide) and R9-chitosan (without the spacer arm)^61^. While thus promising for a range of indications and nanoparticulate drug delivery systems, the present investigation demonstrates that cell internalization of arginine-rich peptides can be further facilitated through end-tagging with oligotryptophan residues.

Finally, we note that much remains unclear regarding the effects of cationic amphiphilic peptides on cancer cells. For example, as discussed by Kuroda *et al.*, human LL-37 interacts with various reporters in carcinogenesis, such as epidermal growth factor receptor, ERBb2, and FPR2. At the same time, LL-37 (and variants thereof) shows anticancer activity in various cancer cell lines. Moreover, LL-37 is overexpressed in lung, ovarian, and breast cancers, but simultaneously suppresses tumorigenesis in gastric cancer^62^. These and other findings demonstrate a complexity of the effects of cationic amphiphilic peptides on cancer cells, which extend beyond composition-dependent membrane interactions. Nevertheless, the composition-driven differences in peptide-induced membrane stability between cancer cells and non-malignant cells are clearly a key initial step, setting limits for both efficiency and selectivity. They also form the basis for targeted uptake of peptide-coated nanoparticulate drug delivery systems and combinations between membrane-active peptides and conventional low Mw cancer therapeutics for reaching increased uptake and efficiency.

## Conclusions

Oligotryptophan end-tagging provides a powerful way to increase internalization of arginine-rich peptides into melanoma cells, but does not enhance peptide uptake in non-malignant cells such as fibroblasts and keratinocytes. This, in turn, results in selective toxicity against melanoma cells, related to both apoptosis and necrosis. These effects correlate to increased peptide adsorption to, and destabilization of, the outer membrane in melanoma cells, caused by the presence of anionic lipids such as phosphatidylserine and ganglioside GM1, as well as to mitochondria membranes. In addition, W-tagging of such peptides offers an approach for targeting of nanoparticle drug carriers to melanoma, and also to facilitate uptake of low Mw anticancer drugs and provide synergistic anticancer effects, here demonstrated for doxorubicin. Thus, W-tagged arginine-rich peptides offer not only direct anticancer effects, but also adjuvant (i.e., facilitation of anticancer effects of other anticancer therapeutics), and targeting possibilities for drug nanocarriers. Notably, these effects were all demonstrated for a very short peptide sequence, which translates into advantages related to both cost of goods and chemical stability in a drug development context. As previously demonstrated for antimicrobial effects^16^, W-tagging is expected to be a generally applicable approach for increasing both potency and selectivity of anticancer peptides.

## Additional Information

**How to cite this article**: Duong, D. T. *et al.* Pronounced peptide selectivity for melanoma through tryptophan end-tagging. *Sci. Rep.* **6**, 24952; doi: 10.1038/srep24952 (2016).

## Supplementary Material

Supplementary Information
