# Supplementary material for: Pronounced peptide selectivity for melanoma through tryptophan end-tagging
Source: Sci Rep. 2016 Apr 27;6:24952. doi: 10.1038/srep24952 (PMC4847013; doi:10.1038/srep24952)

Supporting Material

Pronounced peptide selectivity for melanoma through tryptophan end-tagging

Dinh Thuy Duong^1^, Shalini Singh^2^, Mojtaba Bagheri^2,+^, Navin Kumar Verma^1^, Artur Schmidtchen^1,3^, and Martin Malmsten^2,*^

^1^Lee Kong Chian School of Medicine, Nanyang Technological University, 11 Mandalay Road, Singapore 308232

^2^Department of Pharmacy, Uppsala University, SE-75123, Uppsala, Sweden

^3^Division of Dermatology and Venereology, Department of Clinical Sciences, Lund University, SE-221 84 Lund, Sweden

^+^Present address: Peptide Chemistry Laboratory, Institute of Biochemistry and Biophysics, University of Tehran, 1417614335 Tehran, Iran

^*^Corresponding author. Tel: +46184714334; Fax: +46184714377; E-mail: martin.malmsten@farmaci.uu.se

Key words: anticancer, antimicrobial peptide, melanoma, membrane

**Figure S1.** (a) Particle size distribution of DOPC/GM1 (1/1 mol/mol) liposomes and (b) mean particle size of the indicated liposomes in the absence and presence of 1 μM GRR10W4 in 10 mM Tris, pH 7.4.

(a)

(b)

**Figure S2.** Circular dichroism spectra of GRR10W4N in 10 mM Tris buffer, pH 7.4, and the absence or presence of liposomes at different composition.

(a)

(b)

(c)

**Figure S3.** (a) Representative confocal microscopy images of the time-dependence of GRR10W4 uptake in RPMI 7951 melanoma cells. For this, cells were incubated with 10 μM Alexa488-conjugated peptide for the indicated times.


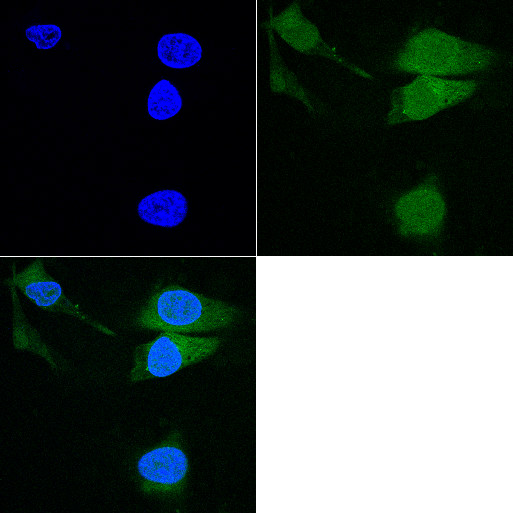

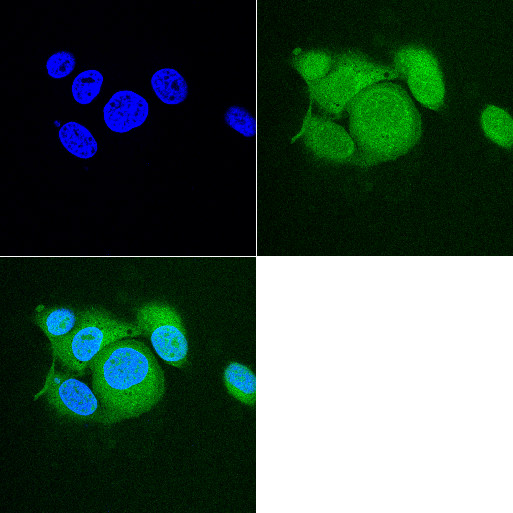

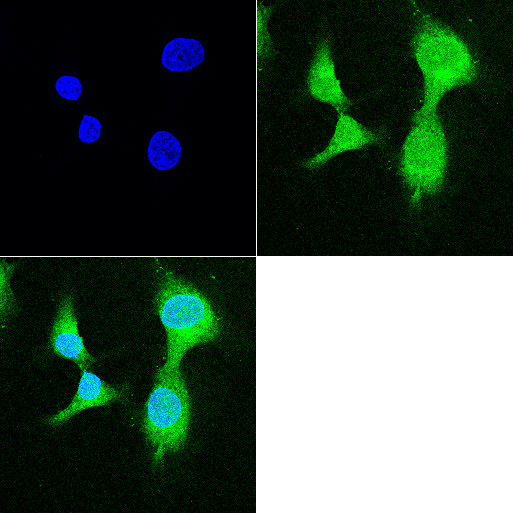


15 min 30 min 120 min

**Figure S4.** Effect of temperature on uptake of GRR10W4 in RPMI 7951 (a) and MeWo (b) cells, demonstrating inactivation at low temperature. Peptides were incubated with the cells at 5 μM for 2 h at either 37^o^C (left) or 4^o^C (right).


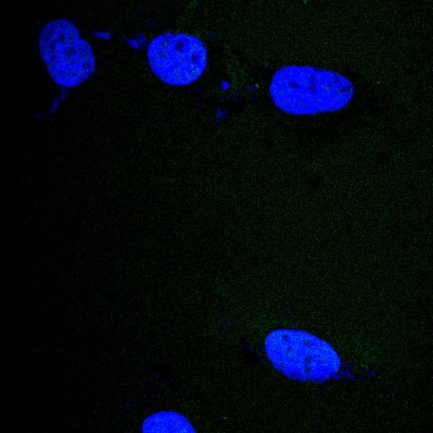

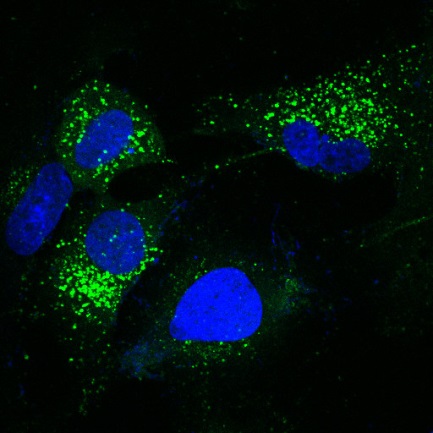
(a)

37^o^C 4^o^C

(b)


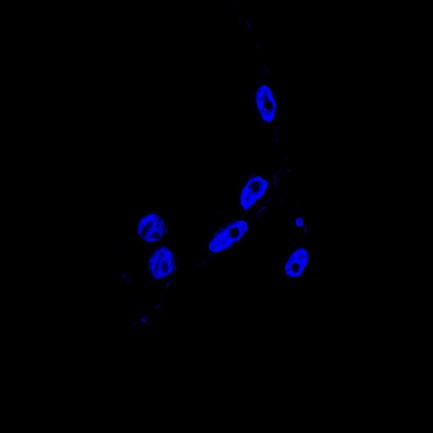

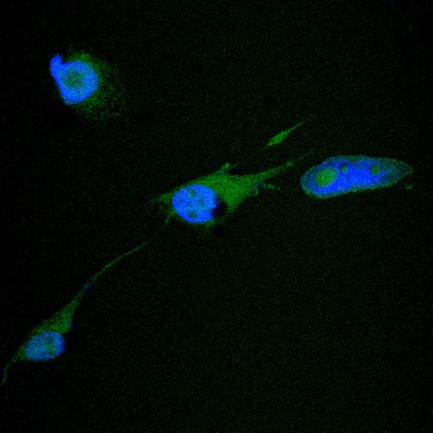


37^o^C 4^o^C

**Figure S5.** (a) Flow cytometry results on peptide toxicity against MeWo melanoma. Cells were incubated with peptides at the indicated concentrations for 48 h, followed by staining with annexin-V and PI and flow cytometric analysis. (b) Quantification of the fraction of dead cells after peptide exposure.

(a)
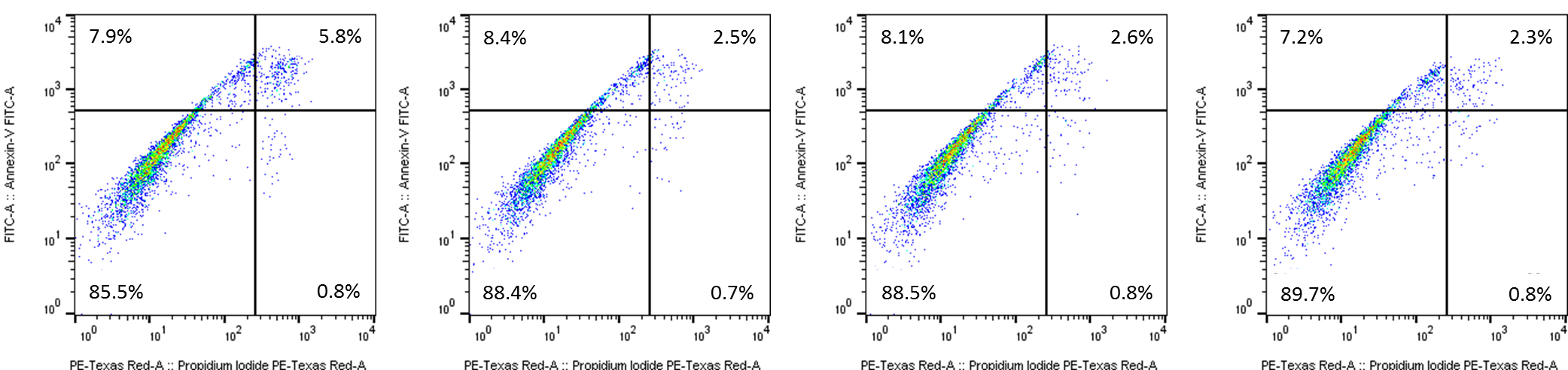


No treatment GRR10 (10 μM) GRR10 (25 μM) GRR10 (50 μM)


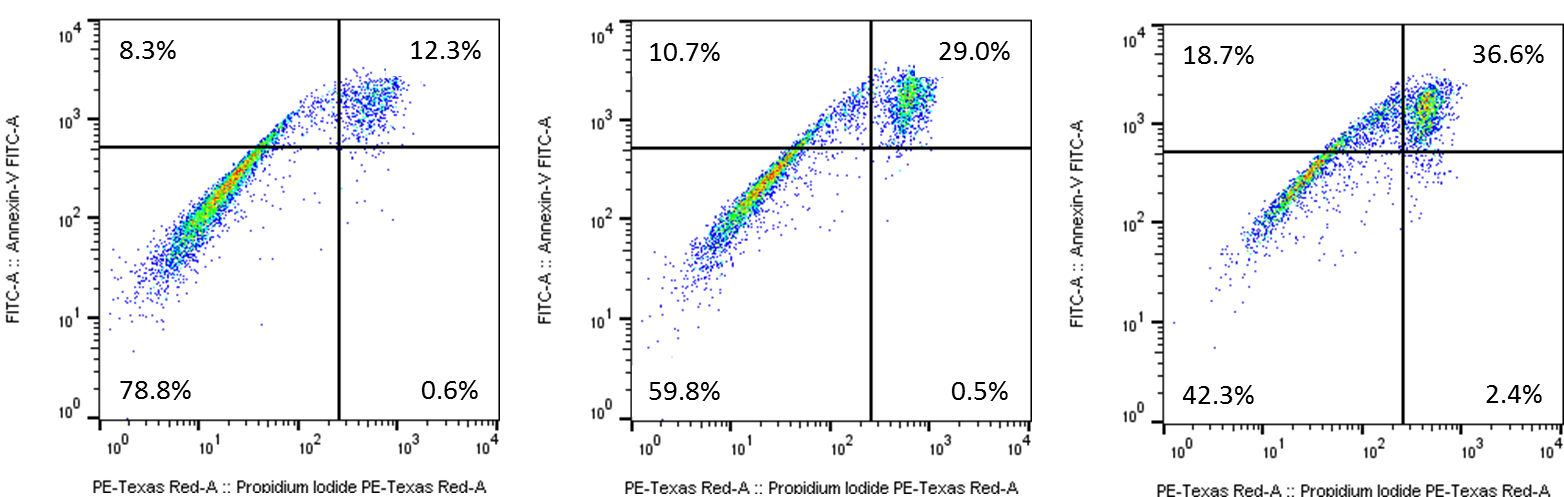


GRR10W4 (10 μM) GRR10W4 (25 μM) GRR10W4 (50 μM)

(b)

**Figure S6.** Toxicity of GRR10 and GRR10W4 on MeWo melanoma. Cells were exposed to the peptides at the indicated concentrations for 48 h in either 1 or 10% FBS, whereafter cell toxicity was monitored by MTT assay. As seen, GRR10W4, but not GRR10, reduces cell survival, most clearly seen at 50 μM.


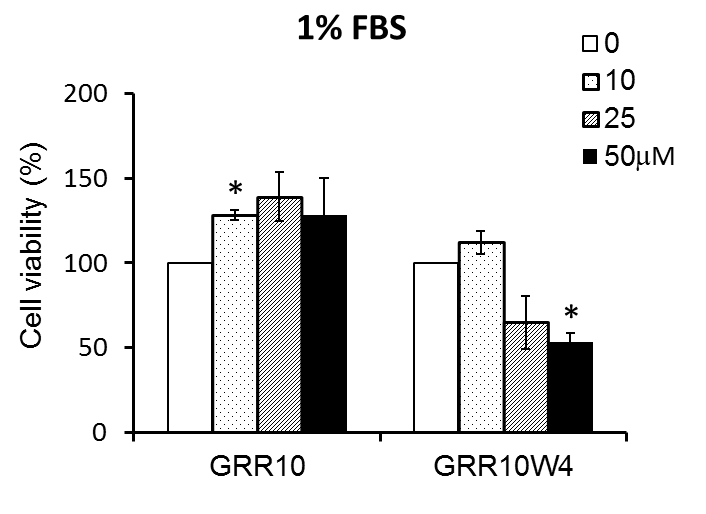

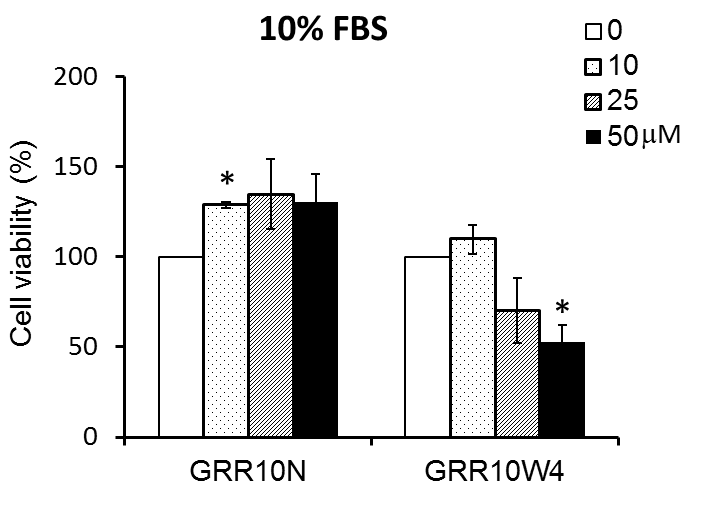

Supplement: Supplementary Information [file srep24952-s1.docx]
